# Suboptimal outcomes and treatment burden of anti-vascular endothelial growth factor treatment for diabetic macular oedema in phakic patients

**DOI:** 10.1038/s41433-023-02667-w

**Published:** 2023-08-04

**Authors:** Christina Rennie, Andrew Lotery, Jo Payne, Moushmi Singh, Faruque Ghanchi

**Affiliations:** 1 University Hospital Southampton NHS Foundation Trust, Southampton, UK; 2 Faculty of Medicine, University of Southampton, Southampton, UK; 3 AbbVie Ltd, AbbVie House, Vanwall Business Park, Maidenhead, UK; 4 Bradford Teaching Hospitals NHS Foundation Trust, Bradford, UK

**Keywords:** Health care, Vision disorders

## Abstract

**Objectives:**

In England and Wales, treatment options were limited for patients with diabetic macular oedema (DMO) with phakic eyes that failed anti-vascular endothelial growth factor (anti-VEGF) treatment pre-2022. This study aimed to quantify the response to, and treatment burden of, anti-VEGF treatment in phakic eyes.

**Methods:**

Retrospective, cohort study using electronic patient record data from two UK centres between 2015 and 2020. Primary objective was proportion of phakic eyes with a suboptimal response after initial 6 months of anti-VEGF treatment. Data were available for 500 eyes from 399 patients.

**Results:**

At 6 months significantly more eyes had a suboptimal response to anti-VEGF treatment: 65.8% (95% CI 61.5–70.0%) vs 34.2% (95% CI 30.0–38.5%), *p* < 0.0001. Baseline visual acuity (VA) predicted VA outcome, however, despite greater gains in eyes with poorer VA, such eyes did not achieve the same VA levels as those who started treatment with better VA. Only 53.6% of eyes had more than three injections in the first 6 months indicating difficulties in delivering high volume/high frequency treatment. Treatment and review burden were similar over the following years regardless of response to anti-VEGF treatment.

**Conclusions:**

Data confirm previous real world evidence around response to anti-VEGF treatment, importance of baseline VA and frequency of injections in predicting outcomes in a UK setting. Continuing treatment beyond 6 months in suboptimal responders imposes unnecessary treatment burden without significant change in VA. In suboptimal responders, consideration of early switch to longer acting steroid treatments may help to reduce treatment burden, whilst maintaining or improving vision.

## Introduction

In the UK diagnoses of diabetes have doubled over the past 15 years, and there are currently almost 4.1 million people in the UK with either type 1 or type 2 diabetes, predicted to rise to 5.5 million by 2030 [1]. Around 6.8% of people with diabetes have diabetic macular oedema (DMO) [2], with prevalence considerably higher in people with type 1 diabetes than type 2. It can be estimated that there are around 279,000 people with DMO in the UK.

DMO is the major cause of vision loss in people with diabetes [3] and has a considerable impact on quality of life. Loss of visual acuity (VA) is correlated with central macular involvement, in particular central macular thickening [4]. The goal of treatment is to preserve or improve retinal function and vision by reducing retinal thickening and oedema [5].

Angiogenesis, increased vascular permeability and inflammation all play a role in the development of DMO [5]. Inflammatory mediators including vascular endothelial growth factor (VEGF) are increased, resulting in increased permeability of the endothelial cells in the retina.

Control of blood pressure, glycaemia and lipids is fundamental in people with diabetes, reducing the risk of development and worsening of diabetic complications, including retinopathy. Current standard of care for DMO in the UK is to reduce macular oedema using intravitreal anti-vascular endothelial growth factor (anti-VEGF) agents if central retinal thickness (CRT) is ≥400 µm [5, 6]. Currently, ranibizumab or aflibercept are the anti-VEGF agents of choice, though faricimab was approved for the treatment of DMO in 2022 [7–9].

Response rates to anti-VEGF agents vary in the literature due to differences in the definition of insufficient response, however it is clear that a significant proportion of patients have a suboptimal response to anti-VEGF treatment [5, 6]. Until recently, treatment options were limited for these patients in England and Wales due to restrictions by the National Institute for Health and Care Excellence (NICE), who restricted intravitreal corticosteroids (dexamethasone implant [OZURDEX] and fluocinolone acetonide implant [ILUVIEN]) to use in an eye with an intraocular (pseudophakic) lens where DMO does not respond to non-corticosteroid treatment, or such treatment is unsuitable [10, 11]. Therefore, people with phakic eyes had limited second-line treatment options. More recently in 2022, NICE recommended dexamethasone intravitreal implant for people with DMO, when their condition has not responded well enough to, or they cannot have non-corticosteroid treatments, which gives patients with a suboptimal response to anti-VEGF treatment an alternative treatment option [12].

Intravitreal corticosteroids for DMO are supported by a strong evidence base. Dexamethasone implant offers efficacy for up to approximately 6 months and is supported by long-term data from randomised controlled trials including the MEAD study [13–17] and by real world evidence [18–21]. Fluocinolone acetonide implant offers efficacy for up to approximately 3 years is also supported by long-term data from the randomised controlled FAME study [17, 22–24] and by real world evidence, much of which is from the UK [25–33].

Corticosteroid implants may result in raised intraocular pressure (IOP) in some patients, particularly in those with a relatively high baseline IOP, a previous IOP rise or a history of glaucoma, which is reflected in increased monitoring to detect any changes in IOP in a timely fashion [34]. Real world evidence has demonstrated that raised IOP can usually be manged with topical medication, with surgery being infrequently required [28, 30, 35, 36].

The aim of this study is to understand the response to treatment in phakic eyes undergoing anti-VEGF treatment for DMO, together with the treatment burden and the unmet need in phakic patients who are unable to access second-line intravitreal corticosteroids.

## Materials/subjects and methods

The lead clinician and Caldicott Guardian at each centre gave written approval for extraction of anonymised data. The study protocol was approved by the lead clinical centre (Southampton). The study was conducted in accordance with the Declaration of Helsinki and the UK Data Protection Act.

This retrospective, multi-centre cohort study included phakic eyes with DMO from two UK centres (Southampton and Bradford). Data from electronic patient records (Medisoft) were extracted anonymously for eyes that had received anti-VEGF injections for DMO in the 5-year period 2015 to March 2020. Best recorded visual acuity (BRVA), OCT, demographic, ocular history and treatment burden data were analysed. The BRVA was converted to Early Treatment Diabetic Retinopathy Study (ETDRS) from logMAR or Snellen recording.

Eyes were included if they were phakic at start of anti-VEGF treatment, treated with aflibercept or ranibizumab and aged over 18 years. Eyes were excluded if they were pseudophakic at baseline, treated with bevacizumab, had macular oedema due to other causes e.g. retinal vein occlusion, treatment started after 31/12/2019 or received intravitreal steroid in the 6 months (dexamethasone implant) or 3 years (fluocinolone acetonide implant) prior to starting anti-VEGF treatment.

## Analysis

The primary objective of the study was to determine the proportion of phakic eyes with DMO with a suboptimal response after the initial 6 months of anti-VEGF injections. Data were obtained for 3 months and 6 months treatment. Six months was chosen for the primary objective as per recent Consensus guidelines published by Downey et al. in [5], which recommend that anti-VEGF therapy should be assessed after the initial six monthly injections and a change in therapy considered, furthermore more data were available for the 6-month dataset making the statistical analysis more robust. A suboptimal response was defined as ≤5 letter gain or <20% reduction in central subfield thickness (CST) as per the study by Busch et al. of continued anti-VEGF treatment vs early switch to dexamethasone [37, 38].

Secondary objectives were to determine the ongoing treatment burden (clinic visits and injections) additional treatments required and rates of cataract and cataract surgery.

All variables were summarised using standard descriptive statistics.

For the primary objective, exact 95% confidence intervals (CI) (two-sided) were computed to express uncertainty in the estimated sample proportion, using the Clopper-Pearson method [39]. A corresponding two-sided test was conducted of the null being that the proportion was 0.5 (i.e., equal suboptimal and optimal proportions).

Proportions were compared, e.g., comparing eyes with good vision (>70 letters) at baseline between the optimal and suboptimal response groups, via Pearson’s chi-squared (two-sided) tests of two independent proportions, with Yates’ continuity correction applied. Corresponding 95% Wald-type CI (two-sided) were also computed to express the uncertainty in the estimated sample difference in proportions.

For VA and OCT summaries over time, 95% Wald-type CI (two-sided) for the mean were computed, using the t-distribution to calculate the critical value to allow for reduced sample sizes at later follow-up time points. Comparisons between mean values were made via independent (two-sided) two-sample t-tests.

For treatment burden data, where multiple clinic visits were recorded on the same day for a patient (in the patient-level encounter data), these were treated as a single visit.

All analyses were performed in the statistical software package R version 3.5.1 with Microsoft R Open 3.5.1.

Further information is available in the Supplement: additional information on statistical methods.

## Results

**Fig. 1:**
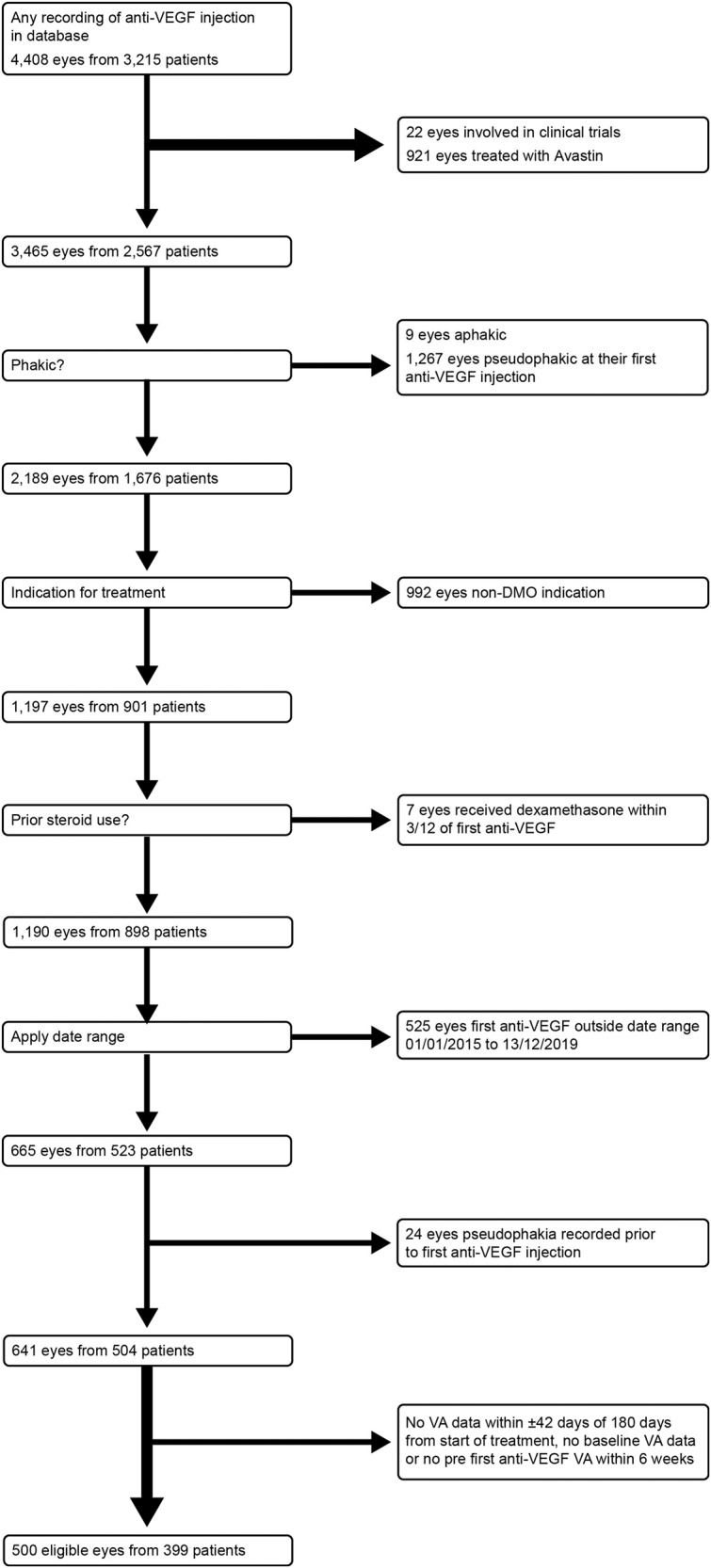
Patient recruitment into the study.

Five hundred eyes of 399 patients met the study inclusion criteria (Fig. 1).

Table 1 summarises baseline demographics. Mean age for starting anti-VEGF treatment for DMO was 61.5 years, with duration of diabetes at start of treatment being longer in patients with type 1 than type 2 diabetes (20.8 years versus 14.1 years).

**Table 1:** Demographic data.

	Full cohort 500 eyes /399 patients	Optimal 171 eyes/153 patients	Suboptimal 329 eyes/285 patients
Age (years) at first anti-VEGF treatment (patients)			
Mean ± SD	61.5 ± 13.5	59.3 ± 13.6	62.6 ± 13.2
Range	21.7–94.7	25.1–94.7	21.7–90.3
Male gender, no (%)	261 (65.4%)	94 (61.4%)	194 (68.1%)
BRVA at baseline (mean ETDRS)	64.3 letters	56.2 letters	68.5 letters
Diabetes type 1/2/unknown			
No	53/319/27	22/119/12	41/229/15
%	13.3/79.9/6.8%	14.4/77.8/7.8%	14.4/80.4/5.3%
Duration of diabetes at first anti-VEGF treatment (first eye), years			
All	15.24 ± 11.01	15.24 ± 10.61	15.47 ± 11.55
Type 1	20.83 ± 8.84	18.95 ± 9.67	21.18 ± 7.66
Type 2	14.14 ± 11.12	14.38 ± 10.72	14.32 ± 11.92
Ethnicity, no (%)			
White British/other	306 (76.7%)	103 (67.3%)	229 (80.4%)
Asian (Bangladeshi, Indian, Pakistani)	43 (10.8%)	19 (12.4%)	27 (9.5%)
Other Asian	5 (1.3%)	3 (2%)	4 (1.4%)
Chinese	1 (0.3%)	1 (0.7%)	0 (0%)
Black	8 (2%)	5 (3.3%)	4 (1.5%)
Mixed	3 (0.8%)	2 (1.3%)	2 (0.7%)
Other	6 (1.5%)	4 (2.6%)	4 (1.4%)
Not stated	27 (6.8%)	16 (10.5%)	15 (5.3%)
Baseline medical conditions, no (%)			
Renal failure	6/377 (1.6%)	2/145 (1.4%)	4/269 1.5%
Hypertension	101/377 (26.8%)	32/145 (22.1%)	71/269 (26.4%)
Ophthalmic history (per eyes), no (%)			
Cataract	359 (71.8%)	119 (69.6%)	240 (72.9%)
Glaucoma	10 (2%)	5 (2.9%)	5 (1.5%)
Previous panretinal photocoagulation (PRP)	92(18.4%)	36 (21.1%)	56 (17%)
Previous mac laser	160 (32%)	47 (27.5%)	113 (34.3%)
Previous intravitreal injections (IVI)	2 (0.4%)	0 (0%)	2 (0.6%)
Previous vitrectomy	7 (1.4%)	2 (1.2%)	5 (1.8%)

The number of patients in optimal and suboptimal groups does not equal 399 because some patients have both eyes treated with some falling into each category.

### Primary objective: response to anti-VEGF treatment

At 6 months significantly more eyes achieved a suboptimal response to anti-VEGF than achieved an optimal response: 65.8% (95% CI 61.5–70.0%) vs 34.2% (95% CI 30.0–38.5%) (*p* < 0.0001).

Mean BRVA at baseline was 64.3 ETDRS letters for all eyes, 68.5 ETDRS letters for the suboptimal group and 56.2 for the optimal response group.

Those with good vision (>70 letters) were less likely to achieve the >5 letter gain. 11.1% of eyes (19/171) in the optimal group had >70 letters at baseline vs 53.8% of eyes (177/329) in the suboptimal group with >70 letters at baseline, *p* < 0.0001. If we consider a definition of >5 letters improvement and/or VA > 70 letters for optimal response, then response increased to 348/500, 69.6% optimal response and 152/500, 30.4% suboptimal response at 6 months.

### Visual acuity over time

**Fig. 2:**
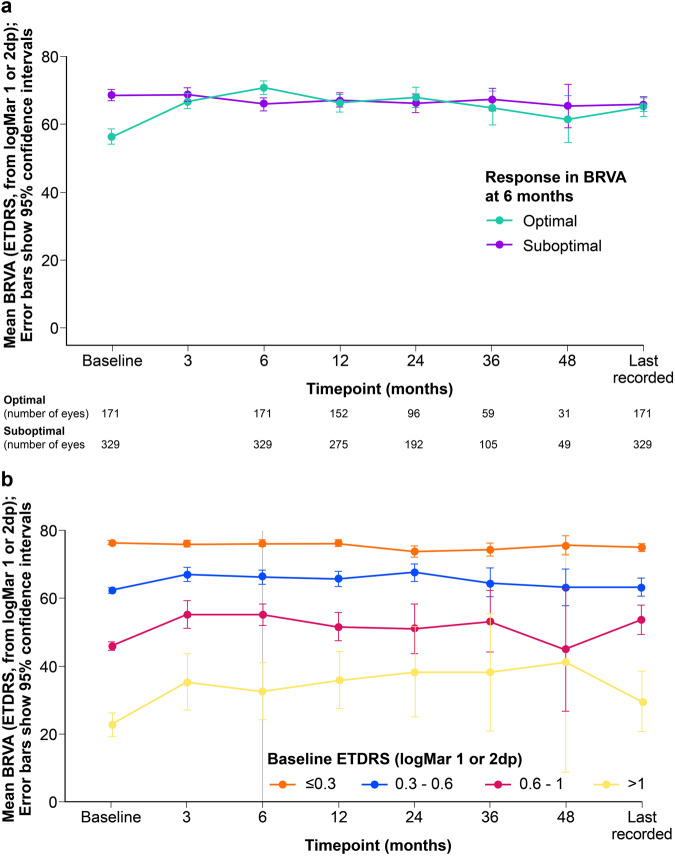
Change in BRVA over time. **a** BRVA over time for optimal and suboptimal groups and **b** BRVA over time according to baseline vision.

Figure 2a shows the change in VA over time for each group. The mean follow-up (time to last recorded VA) was 28.8 months (standard deviation [SD] 15.7) with similar follow up for optimal (29 months) and suboptimal groups (28.8 months). Overall, the mean change in VA was 3.2 EDTRS letters (SD 11.6) at 6 months, with +14.5 letters for the optimal and −2.7 letters for the suboptimal group.

Baseline VA predicts outcome over time (see Fig. 2b). Those in the best VA category (≤0.3 letters, 6/12) have little change and due to good baseline vision are less likely to meet the >5 letter gain.

**Fig. 3:**
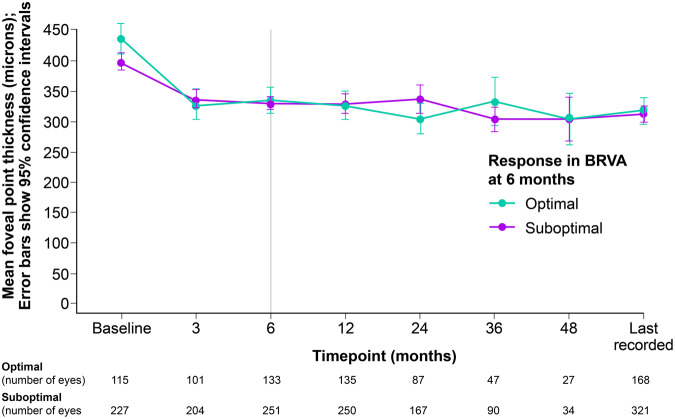
OCT foveal point thickness over time for optimal and suboptimal groups.

There was mean gain from baseline of 3.2 letters (SD 11.6) in BRVA at 6 months, 2.6 letters (SD 12.5) at 12 months, 1.9 letters (SD 15.3) at 24 months and 36 months (SD 15.2) and 1.8 letters (SD 17.7) at 48 months. Those eyes with poorest vision had the greatest VA gain.

Eyes with baseline BRVA < 55 letters had mean gain of 9.3 letters (SD 15), those with BRVA < 55 and >70 letters gained 4 letters (SD12.5) at 6 months. At 6 months 20% of eyes gained >10 letters, 10.2% of eyes lost ≥ 10 letters and 65.8% had suboptimal ≤5 letter gain.

### Optical coherence tomography

OCT data were available for 271/500 eyes at the 6 month timepoint. Based on OCT criteria of <20% improvement 43% of eyes were classified as optimal and 57% as suboptimal (95% CI 50.7%–62.8%). A combination of <20% OCT improvement with BRVA of ≤5 letter gain resulted in 81.9% being classified as suboptimal responders (Fig. 3).

### Outcomes by number of injections

At 6 months, 46.4% (232 eyes) had received ≤3 injections and 38% (190 eyes) had no injection between 3 and 6 months. Of those receiving ≤3 injections 78% (171/232 eyes), all injections were received within the first 3 months of the first injection. Of those who received ≤3 injections 30.6% (71/232 eyes) had an optimal response compared with 37.3% (100/268 eyes) who received >3 injections (*p* = 0.1381).

In the optimal group 56/171 eyes (32.7%) had no injection between 3 and 6 months compared with 134/329 eyes (40.7%) in the suboptimal group (*p* = 0.0996).

### Treatment burden

**Fig. 4:**
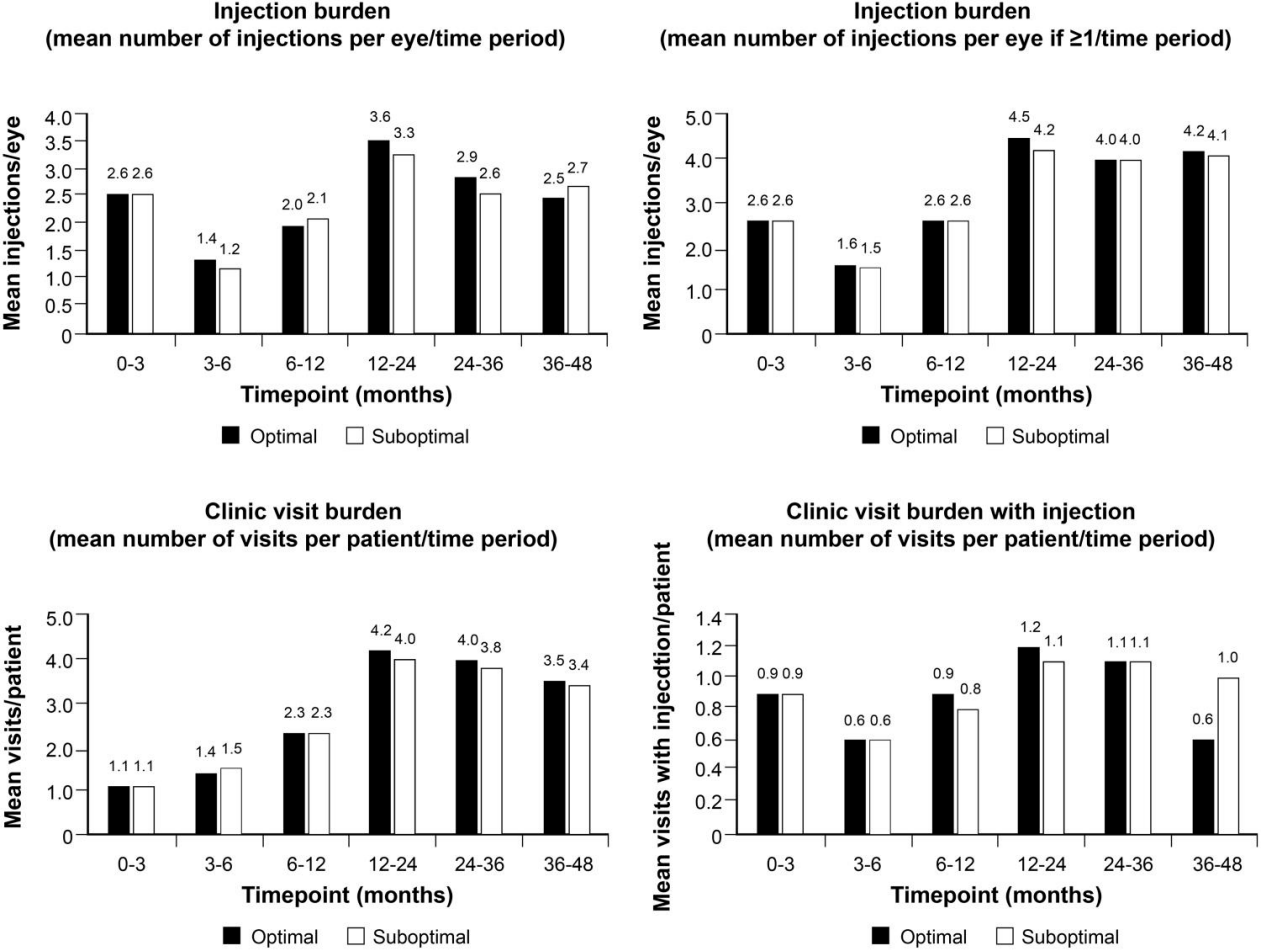
Injection and treatment burden. The top two panels reflect injection burden and the bottom two reflect clinic vist burden.

Figure 4 shows the treatment burden for optimal and suboptimal responding eyes, together with the mean number of clinic visits per patient. In terms of treatment burden, 63% of eyes were still under treatment at 48 months with the optimal group having mean 7.7 letter gain and the suboptimal group −1.9 mean letter gain. Despite the suboptimal response these eyes continued to receive similar number of injections over the follow up period.

For all eyes over the entire follow up period, the mean number of injections was 9.5. Cumulative mean number of injections was 3.7 for the first 6 months, 5.8 for 12 months, 9 for 24 months, 12.1 for 36 months and 15.3 for 48 months.

Mean number of visits to final review for all patients was 9.9 (SD 6.7). Cumulative mean number of clinic visits was 2.4 for the first 6 months, 4.7 for 12 months, 8.7 for 24 months, 12.7 for 36 months and 15.8 for 48 months. Overall, 101 patients had bilateral anti-VEGF treatment for DMO, of whom 23 patients had an optimal response to treatment in one eye and suboptimal in the other.

### Cataract and cataract surgery

At baseline 28.2% (141 eyes) had no cataract and 71.8% (359 eyes) had presence of cataract recorded. At last recorded vision 93% (465 eyes) had cataract recorded. There was no evidence of a difference between the suboptimal group with 72.9% (240 eyes) having cataract recorded at baseline and 69.6% (119 eyes) of optimal responding eyes (p = 0.4922). There was very little surgery in the first 6 months of treatment with 15/500 eyes (3%) undergoing surgery, however, this steadily increased to 6.8% (29/427 eyes with data) by 12 months, 13.9% (40/288 eyes with data) at 24 months, 22.6 % (37/164 eyes with data) at 36 months and 36.2% (29/80 eyes with data) at 48 months. By the last recorded VA 18.2% of all the eyes in the cohort (91/500) had undergone cataract surgery.

### Other treatments during follow-up

A significant number of eyes had received previous treatment for diabetic retinopathy and maculopathy (see Table 1). During the study period 10.8% (54 eyes) received PRP, 5.2% (26 eyes) macular laser, 1.8% (9) vitrectomy surgery and 0.2% (1 eye) glaucoma surgery.

Other intravitreal injections were given in 4.6% (23 eyes) over the follow-up period, dexamethasone implant was given in 4.2% (21 eyes, 29 injections, three during cataract surgery and nine in phakic eyes), fluocinolone acetonide implant was given to 0.6% (three eyes, two psuedophakic and one phakic). Ceftazidime was given in 0.4% (two eyes) for endophthalmitis. The endophthalmitis event per injection was two in 4,962 injections, 0.04% or 0.4 cases per thousand injections given. Post-operative uveitis was only recorded in one eye. The most common post-operative complication was raised IOP with 11 events recorded in seven eyes, giving a 0.22% event rate. Post-operative adverse events rely on recording in clinic so may not be accurate.

## Discussion

Anti-VEGF treatment in DMO patients with phakic eyes maintains or improves vision in the real world, however, in this study 65.8% of phakic eyes had a suboptimal response to anti-VEGF treatment at 6 months. In line with analysis of the results from the randomised clinical trials of anti-VEGF drugs for the treatment of DMO most VA gains were achieved within the initial 3–6 months of therapy [6].

Baseline VA predicts VA outcome; at the 6-month timepoint despite greater gains in eyes with poorer vision patients did not achieve the same VA levels as those who started treatment with better vision.

Definitions of suboptimal response vary between clinical trials [6, 38, 40, 41] and rate of suboptimal response in this cohort is higher than expected. There was a high baseline VA and the BRVA was only available to two decimal places in 59% of cases. Baseline mean BRVA of 64.3 ETDRS letters may limit the amount of visual gain seen, especially for the 53.7% of the suboptimal group who have >70 letters at baseline. There is a need to readjust the definition of response to treatment to reflect real world experience. In clinical practice patients with DMO are often identified early and are able to access treatment promptly with VA of >70 letters. Using a definition for optimal response of >5 letters improvement or a VA > 70 letters with 20 % reduction of fluid on OCT may be a more useful assessment.

Irrespective of baseline VA, patients in England need to have >400 microns of retinal thickness in order to qualify for anti-VEGF treatment [8, 9], meaning that these eyes have significant macular oedema on OCT at baseline. OCT is consistent and often used as the marker for response to treatment as VA can be more variable with difficulties in performance and non-refracted assessments. However, it is well established that there is poor correlation between VA and OCT or CRT [42, 43]. The proportion of suboptimal eyes of 57% based on OCT in this real world evidence is much closer to the clinical trials data than using BRVA alone. Data from the Protocol V study [44] of initial observation vs anti-VEGF treatment, and RIDE and RISE Trials [45], all show that delay in anti-VEGF treatment results in lower VA gains and absolute VA levels compared with prompt anti-VEGF initiation. This supports the treatment of significant DMO despite good vision, as delay may give suboptimal final visual outcomes [46].

Only 53% of eyes had more than three injections in the first 6 months indicating difficulties in delivering high volume and high frequency treatment in clinical practice, which is similar to other real world evidence [47]. It should be noted that 38% of eyes did not receive any injections between 3 and 6 months from initiation of treatment. The number of anti-VEGF injections at 6 months did not significantly impact on whether eyes had an optimal response, although failure to deliver treatment in the second 3 months might lead to a loss of any benefit gained from the initial treatment. However, on initial evaluation of the data there was very little difference in rates of suboptimal response at 3 months vs 6 months. At 3 months: 35.2% (154/437 eyes) had optimal and 64.8% (283/437, 95% CI 60.1–69.2%) had suboptimal response to treatment. Whether eyes had an optimal or suboptimal response, the treatment and review burden were similar over the following years.

A group of UK retina experts have expressed concern that there is no clear guidance for when to consider switching patients with DMO and an insufficient response to anti-VEGF treatment to other alternative treatments such as intravitreal corticosteroid therapy [5]. Data from Protocol I show that for those with poor response at 3 months, 53% continue to have a suboptimal functional response at 3 years [48]. Continuing treatment beyond 6 months therefore imposes a large treatment burden with only modest clinical benefit in suboptimal responders. Indeed, in a retrospective, multicentre, case-control study eyes with DMO with suboptimal response (defined as refractory to anti-VEGF therapy after three monthly injections) had better visual and anatomical outcomes at 12 and 24 months if they were switched to intravitreal corticosteroid treatment at 3 months compared with continued anti-VEGF therapy [37, 38]. A UK Consensus paper recommends switching to corticosteroid to improve efficacy of treatment if there is insufficient response after a maximum of six monthly anti-VEGF treatments, or to reduce treatment burden if at 24 months, ≥6 injections in the preceding 12 months have not resulted in response [5].

This indicates a focus on delivering more treatment within the first 6 months, with consideration that early switch to longer acting steroid treatments at this point may help in reducing treatment burden in suboptimal responders, whilst maintaining or improving vision. These patients will however continue to require some clinic review appointments to monitor and manage other complications such as proliferative retinopathy.

The presence of cataract is common in this group of patients which is consistent with their age and diagnosis of diabetes. Few cataract operations are carried out in the first year which fits with the clinical strategy of stabilising the retina and optimising this before considering cataract surgery. The high prevalence of cataract and subsequent need for surgery may be used to facilitate conversations about treatment switch to steroid implants.

This study has limitations inherent in a retrospective non-interventional study but provides an insight into real-world practice in the UK and indication for considering early switch to steroid treatment. Although this analysis focused on the ongoing outcomes and treatment burden based on response at 6 months, rather than number of injections, it would also have been valuable to consider outcomes in those patients who received the recommended number of five loading dose injections in the first 6 months of treatment. This would help to determine whether, as the number of responders increased, the subsequent injection burden decreased. Unfortunately, use of real world data meant that the sample size was relatively small (*n* = 108) making it challenging to draw conclusions.

In conclusion anti-VEGF treatment for DMO patients with phakic eyes in real-world UK clinical practice can maintain or improve vision. Although outcomes are poorer than clinical trials, it is consistent with other real world evidence [47]. At 6 months 65.8% of phakic eyes had a suboptimal response to anti-VEGF treatment. Those with poorer baseline BRVA had greater gains at 6 months however, baseline VA predicts the VA outcome, so despite the greater gains in letters these eyes do not achieve the same VA levels as those who start with better vision. High baseline VA is a reflection of early detection and prompt treatment, and VA is maintained which is important for functional outcomes.

Beyond 6 months, optimal and suboptimal responders had similar ongoing treatment and review burden over 4 years of anti-VEGF treatment. It is recognised that clinicians would have adjusted their decision about retreatment and/or treatment intervals based on the absence of an increase in macular oedema. Thus, for the suboptimal responders this treatment helped to maintain some VA, but consideration should be given to early switch to longer acting treatments to reduce treatment burden and improve outcomes.

### Data sharing

AbbVie is committed to responsible data sharing regarding the clinical trials we sponsor. This includes access to anonymised, individual and trial-level data (analysis data sets), as well as other information (e.g., protocols, clinical study reports, or analysis plans), as long as the trials are not part of an ongoing or planned regulatory submission. This includes requests for clinical trial data for unlicensed products and indications.

These clinical trial data can be requested by any qualified researchers who engage in rigorous, independent, scientific research, and will be provided following review and approval of a research proposal, Statistical Analysis Plan (SAP), and execution of a Data Sharing Agreement (DSA). Data requests can be submitted at any time after approval in the US and Europe and after acceptance of this manuscript for publication. The data will be accessible for 12 months, with possible extensions considered. For more information on the process or to submit a request, visit the following link: https://www.abbvie.com/our-science/clinical-trials/clinical-trials-data-and-information-sharing/data-and-information-sharing-with-qualified-researchers.html

## Summary

### What was known before

- Retrospective real world studies of anti-VEGF treatments have reported lower injection frequency and functional gains than randomised clinical trials.
- Improvement of functional and anatomical parameters in response to anti-VEGF treatment is closely associated with baseline BRVA.

### What this study adds

- Confirms previous real world evidence around frequency of injections, response to anti-VEGF treatment and importance of baseline BRVA in predicting outcomes in a UK setting.
- Continuing treatment beyond 6 months in patients with a suboptimal response imposes a large treatment burden with only modest clinical benefit.
- High prevalence of cataract and subsequent need for cataract surgery may help facilitate conversations about switch to steroid use.

### Supplementary information

Supplement: additional information on statistical methods
