# Supplementary material for: Suboptimal outcomes and treatment burden of anti-vascular endothelial growth factor treatment for diabetic macular oedema in phakic patients
Source: Eye (Lond). 2023 Aug 4;38(1):215–23. doi: 10.1038/s41433-023-02667-w (PMC10764926; doi:10.1038/s41433-023-02667-w)
Supplement: Supplementary file 1 — Supplement: additional information on statistical methods [file 41433_2023_2667_MOESM1_ESM.docx]

**Sub-optimal outcomes and treatment burden of anti-vascular endothelial growth factor treatment for diabetic macular oedema in phakic patients**

# Supplement: additional information on statistical methods

The following time-windows were used for the various analysis time points, based on VA or optical coherence tomography (OCT) data available for each eye.

- Baseline: Closest encounter date (on or) within 42 days prior (i.e., -6 weeks) to first anti-VEGF injection.
- 3 months: Closest encounter date to 90 days (i.e., 3 months) after date of first anti-VEGF injection up to 14 days prior or 28 days post (i.e., -2 weeks & +4 weeks).
- 6 months: Closest encounter date to 180 days (i.e., 6 months) after date of first anti-VEGF injection up to ±42 days (i.e., ±6 weeks).
- 12 months: Closest encounter date to 365 days (i.e., 1 year), to up to ±90 days (i.e., ±3 months).
- 24 months: Closest encounter date to 730 days (i.e., 2 years), to up to ±90 days (i.e., ±3 months).
- 36 months: Closest encounter date to 1095 days (i.e., 3 years), to up to ±90 days (i.e., ±3 months).
- 48 months: Closest encounter date to 1460 days (i.e., 4 years), to up to ±90 days (i.e., ±3 months).

Some eyes had multiple foveal point thickness results on the same day, across repeated OCT scans (this occurred for just under 10% of the complete OCT results extracted). In these cases, the thickest value recorded was used in the analysis. OCT results were from Topcon machines adjusted to compare with the Heidelberg results as follows: Topcon 2000 conversion +50 microns^1, 2^, DRI OCT SS conversion for CPT 36.55+0.988xSSOCT^3^.

All variables were summarised using standard descriptive statistics. For continuous variables, the number of eyes and/or patients (n), number of missing values, mean, standard deviation (SD), median, lower quartile (Q1), upper quartile (Q3), minimum (Min.), and maximum (Max.) value were calculated. For categorical variables, the number of missing values, counts and percentages (out of all values, and out of the non-missing values) of the eyes and/or patients in each category were calculated.

# References

1. Tan CS, Chan JC, Cheong KX, Ngo WK, Sadda SR. Comparison of retinal thicknesses measured using swept-source and spectral-domain optical coherence tomography devices. *Ophthalmic Surg Lasers Imaging Retina* 2015; **46**(2)**:** 172-179.

2. Mylonas G, Ahlers C, Malamos P, Golbaz I, Deak G, Schuetze C *et al.* Comparison of retinal thickness measurements and segmentation performance of four different spectral and time domain OCT devices in neovascular age-related macular degeneration. *Br J Ophthalmol* 2009; **93**(11)**:** 1453-1460.

3. Xiong K, Gong X, Li W, Yuting L, Meng J, Wang L *et al.* Comparison of Macular Thickness Measurements Using Swept-Source and Spectral-Domain Optical Coherence Tomography in Healthy and Diabetic Subjects. *Curr Eye Res* 2021; **46**(10)**:** 1567-1573.
